# Rare Cases of Bronchial Aneurysm and Comparison of Interventional Embolization in the Treatment of True Bronchial Aneurysm and Pseudobronchial Aneurysm

**DOI:** 10.3389/fcvm.2022.856684

**Published:** 2022-03-09

**Authors:** Jia-Li Lin, Yuan-Yuan Ji, Ming-Zhe Zhang, Yi Tang, Ruo-Li Wang, Dan-Dan Ruan, Yan-Feng Zhou, Shao-Jie Wu, Sen-Lin Cai, Jian-Hui Zhang, Xiao-Rong Meng, Jie-Wei Luo, Zhu-Ting Fang

**Affiliations:** ^1^Shengli Clinical Medical College of Fujian Medical University, Fuzhou, China; ^2^Department of Interventional Radiology, Fujian Provincial Hospital, Fuzhou, China

**Keywords:** bronchial artery aneurysm, transcatheter artery embolization, pseudobronchial aneurysm, endovascular treatment, rare disease

## Abstract

**Background:**

Bronchial artery aneurysm (BAA) is a rare disease. Rupture of BAA can lead to life-threatening hemoptysis, and once diagnosed, treatment is needed regardless of symptoms. Transcatheter artery embolization is the first choice of treatment because it is minimally invasive and effective. This study aimed to retrospectively compare the embolization treatment of a case of true BAA and that of a pseudobranchial aneurysm and explore the choice of embolization method for BAA with short neck or no neck.

**Materials and Methods:**

Embolization treatment and imaging characteristics of one case of true BAA and one case of pseudobronchial aneurysm admitted to our hospital were analyzed retrospectively. Embolization methods and therapeutic effects of two cases of BAAs were compared.

**Results:**

Case 1 was that of an intact true BAA inside the mediastinum located at the opening of the bronchial artery. The distal end of the aneurysm was embolized, and tumor cavity was occluded. No recurrence of BAA was found after the operation. Case 2 was that of a ruptured and hemorrhagic pseudobronchial aneurysm of the mediastinum. Coil embolization combined with covered stent graft exclusion of the thoracic aorta were performed, and the left bronchial artery and BAA were almost occluded. Nine months postoperatively, the mediastinal hematoma was almost completely absorbed.

**Conclusion:**

Endovascular embolization has become the most commonly used for the treatment of BAA. Different methods should be selected according to the location and nature of the aneurysm.

## Introduction

Bronchial artery aneurysm (BAA) is a rare disease, and less than 1% of all patients with selective bronchial arteriography have been found (1). Since the first report of BAA, approximately 60 cases have been reported (2). Rupture of BAA can lead to life-threatening hemoptysis (3), and once diagnosed, treatment is needed regardless of symptoms. Transcatheter artery embolization (TAE) is the first choice of treatment because it is minimally invasive and effective. However, due to its low incidence, there are fewer reports on TAE of BAA in China, we present the embolization methods of two patients with BAAs in our study.

## Case Presentation

### Case 1

**FIGURE 1:**
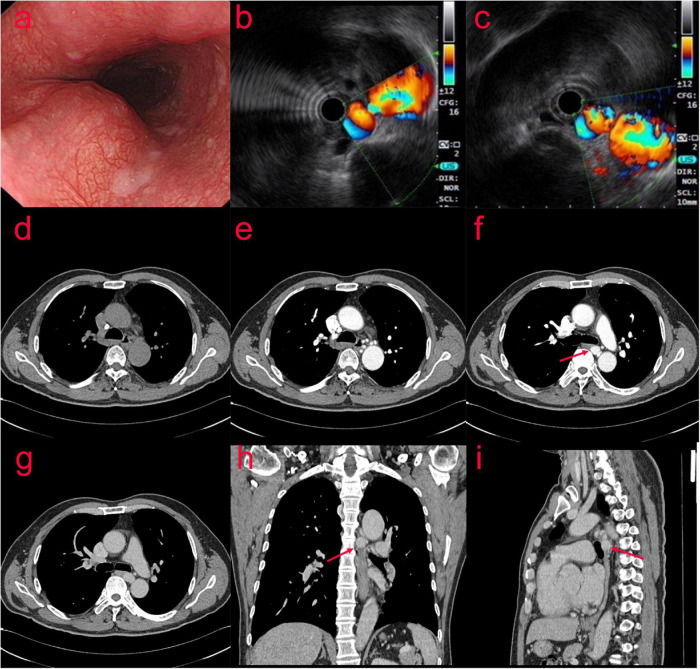
(Case one) The patient with Bronchial artery aneurysm (BAA) was examined by gastroscopy **(a)** at the local hospital, which showed an “esophageal mass.” After visiting our hospital, ultrasonic gastroscopy showed an esophageal bulge, vascular compression, and suspected BAA **(b,c)**. A contrast-enhanced CT scan showed multiple tortuous and thickened vascular shadows (maximum width about 1.3 cm) in the middle and posterior mediastinum and around the esophagus **(d–i)**.

In July 2020, a 63-year-old man presented with recurrent chronic productive cough for more than 40 years prior to admission, occasionally with blood in the sputum. The patient was asymptomatic for dyspnea, dysphagia, or hemoptysis, and denied previous thoracic trauma. Plain chest computed tomography (CT) scan was performed due to cough and shortness of breath with the following findings: bronchiectasis with infection in the middle lobe of the right lung, the lingual segment of the upper lobe, and the lower lobe of the left lung. The patient was examined by gastroscopy (Figure 1a) at the local hospital, which showed an “esophageal mass.” Then, the patient visited our hospital, and ultrasonic gastroscopy showed an esophageal bulge, vascular compression, and suspected BAA (Figures 1b,c). A contrast-enhanced CT scan showed multiple tortuous and thickened vascular shadows, partial aneurysmal dilation (maximum width about 1.3 cm), vaguely connected with the thoracic aorta and left pulmonary vein, and adjacent esophageal compression. Partial bronchiectasis in both lungs with a small amount of inflammation (Figures 1d–i).

The patient was normal on physical examination. His laboratory results were generally non-significant, except *hemoglobin* (Hb) level was 119 g/L (normal value 120∼165 g/L) and D-Dimer was 2.43 mg/L (normal value 0–0.55 mg/L). The patient is a chronic HBV carrier and his laboratory tests revealed normal liver function.

**FIGURE 2:**
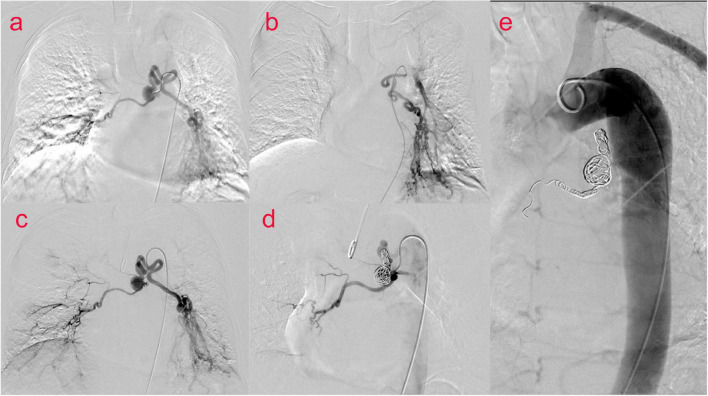
(Case one) Bronchial arteriography **(a–c)** showed an aneurysm at the opening of the bronchial artery of about 1⋅5 cm × 1⋅7 cm. Left and right bronchial artery thickening, disordered vessels in the lung, most obviously in the left lower lung, and pulmonary artery branches were seen. BAA and left bronchial artery and pulmonary artery fistula were considered. Percutaneous catheterization of the right femoral artery thoracic aorta, abdominal aorta, left and right iliac arteries, and left and right bronchial arteries was performed. During digital subtraction angiography (DSA) **(d,e)**, the distal end of the aneurysm was embolized with 700–900 μm microspheres and NESTER coils, and the tumor cavity was occluded.

The patient was then taken to interventional radiology suite for angiographic evaluation. Percutaneous right femoral artery catheterization was performed under local anesthesia. With digital subtraction angiography(DSA), a 5F catheter was used for thoracic aorta and left and right bronchial artery angiography. Bronchial artery angiography showed no obvious abnormality in ascending, arched and descending aorta; It was equivalent to an aneurysm at the opening of the bronchial artery, approximately 1.5 cm × 1.7 cm. The left and right bronchial arteries were thickened, and disorder vessels were seen in the lungs, especially in the lower left lung, with pulmonary artery branches visible. Embolization of the left bronchial artery was performed with gelatin sponge particles (350–560 um), and the blood flow of the left bronchial artery slowed down significantly after embolization. We decided to embolize the BAA and prepare a thoracic aortic stent implantation. Before the procedures he signed a written informed consent to an endoluminal stent of the thoracic aorta in accordance with our institutional guidelines. Percutaneous right femoral artery puncture and catheterization under local anesthesia were performed, and 5F catheters were used for the thoracic aorta, abdominal aorta, left and right iliac arteries, and left and right bronchial arteriography with DSA. The distal end of the aneurysm was embolized with 700–900 μm microspheres and NESTER coils, and the tumor cavity was occluded. To achieve satisfactory angiographic results, no thoracic aortic stent implantation (Figure 2) was performed. The postoperative course was uneventful and the patient was discharged home on postoperative day 6. On CT scan at 4 months, we confirmed satisfactory placement of the aortic stent graft with exclusion and thrombosis of the BAA. No recurrence of bronchial artery aneurysm was found at 10 months after operation.

### Case 2

**FIGURE 3:**
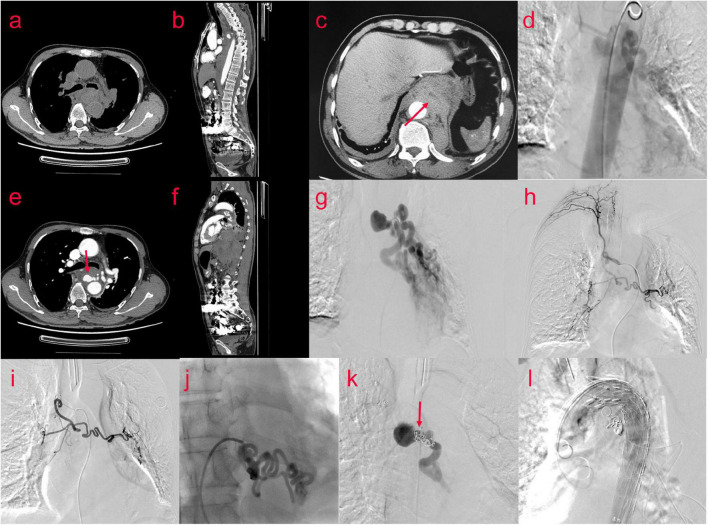
(Case two) A 65-year-old male patient suffered from retrosternal pain for 1 day, the degree was severe, and the scope was diffused. Bronchial arteriography **(d,g,h)** was performed after examination of the chest CTA **(a–c,e,f)**. The aneurysmal dilatation of the proximal end of the left bronchial artery with a diameter of approximately 2.6 cm. Its branches were tortuous and thickened. The left pulmonary artery branches were seen. The right bronchial artery thickened, and the communicating branches were seen between the right bronchial artery and the left bronchial artery. The embolization operation and the thoracic aorta-covered stent implantation were performed. Percutaneous catheterization of left and right femoral artery under general anesthesia and local anesthesia, left and right bronchial artery, and thoracic aorta were performed. Digital subtraction angiography showed that the right bronchial artery was embolized by embolized microspheres (500–700 um) and coils, and the left bronchial artery was embolized by two branches of PVA particles (700 um) **(i,j)**. The left bronchial trunk and proximal segment aneurysms **(k)** were embolized with controllable coils, and a 32/24 × 20 cm covered stent **(l)** was implanted into the aortic arch and descending aorta through the left femoral artery.

A 65-year-old male patient presented to the emergency department with a 1-day history of retrosternal pain, which was located behind the sternum and was diffuse in scope without fixed location and severe in degree, radiating to the xiphoid process, accompanied by shortness of breath and sweating, occasional cough and sputum. He denied any nausea, hematemesis, or hematochezia. His medical history was retinal detachment. Chest computed tomography angiography(CTA) revealed tortuous and thickened arteries in the posterior mediastinum, hilum of the lung, and left lower lung, which were considered abnormally enlarged and dilated bronchial arteries, accompanied by the formation of proximal aneurysms; therefore posterior mediastinal hematoma was likely (Figures 3a–c,e,f). The laboratory results were as follows: peripheral blood white blood cell (WBC) count was 13.8 × 10^9/L (normal value 4–10 × 10^9/L), C reactive protein (CRP) level was 43.70 mg/L (normal value 0.8–8 mg/L), Hb level was 110 g/L, and erythrocyte sedimentation rate (ESR) level was 85 mm in the 1st h. Troponins and D-dimer were negative. Serum tumor markers were negative. Liver and renal function tests were within normal limits. Coagulation profile was normal.

Thoracic surgery and cardiovascular surgery consultation: patients with bronchial pseudoaneurysm, the neck of the aneurysm is shorter, less than 5 mm, the risk of simple embolization is higher, and the patient is prepared for posterior mediastinal hematoma removal in thoracic surgery in the later stage, and the operation has the risk of rebleeding. Therefore, implantation of covered stent in thoracic aorta was proposed. Bronchial arteriography and coil embolization combined with covered stent graft exclusion of thoracic aorta (Figures 3d,g–l). The results showed that aneurysmal dilatation of the proximal end of the left bronchial artery with a diameter of approximately 2.6 cm; its branches were tortuous and thickened, and the left pulmonary artery branches could be seen; the right bronchial artery thickened and communicating branches were seen between the right and left bronchial arteries. Bronchial arteriography and coil embolization combined with covered stent graft exclusion of the thoracic aorta was performed (Figures 3d,g–l). Microspheres were used for embolization (500–700 um) and coils for embolization were used to occlude the right bronchial artery. After embolizing the left bronchial artery with PVA particles, the left main bronchial trunk and proximal segment aneurysms were embolized with controllable coils. After embolization, the malformed vessels of the left bronchial artery decreased significantly, and the left bronchial artery and aneurysm were almost occluded. A 32/24 × 20 cm covered stent was implanted in the aortic arch and descending aorta. Repeated angiography did not reveal a left BAA. The patient was discharged after 3 days in good general condition.

**FIGURE 4:**
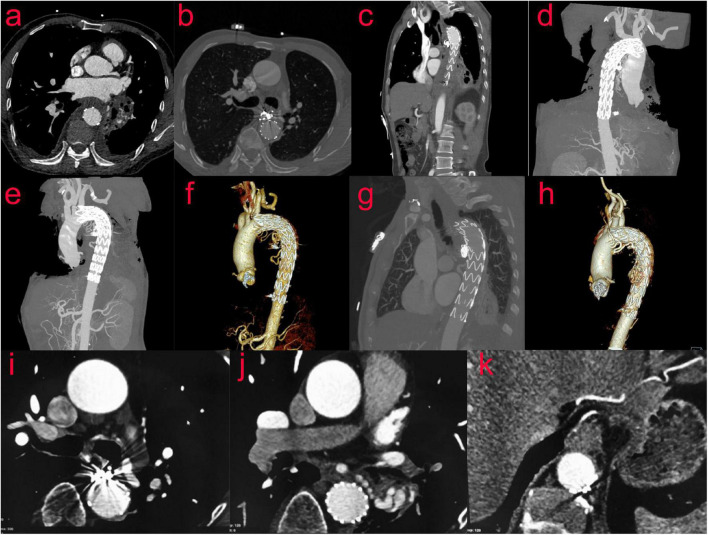
(Case two) CTA of the thoracic aorta was reexamined 10 days after the operation **(a–f)**. After stenting of the aortic arch and descending aorta, the shape and position of the metal stents were evaluated to be good. It adhered to the wall well, and there was no displacement or internal leakage. The mediastinal hematoma was partially absorbed. After 2 months, the reexamination of the thoracic aorta by CTA showed that the mediastinal hematoma was further absorbed, and the stent was in good shape **(g,h)**. Nine months after the operation, the review of CT showed that the mediastinal hematoma was almost completely absorbed **(i–k)**.

CTA of thoracic aorta was reexamined 10 days after operation: after stenting of aortic arch and descending aorta, the shape and position of metal stents were good. It adhered to the wall well, and there was no displacement or internal leakage. The mediastinal hematoma was partially absorbed (Figures 4a–f). A CT scan performed after 2 months showed no endoleak, no stent migration and the mediastinal hematoma was further absorbed. (Figures 4g,h). Nine months after the operation, the mediastinal hematoma was almost completely absorbed (Figures 4i–k).

## Discussion

Bronchial artery aneurysm is a rare entity. However, it can cause life-threatening hemoptysis when it ruptures (3). There are currently no guidelines available in management of BAAs.

Due to the rarity and lack of animal models of BAA, information remains limited regarding its etiology. However, it is often associated with atherosclerosis, inflammatory lung disease, bronchiectasis, bronchitis, and systemic vascular abnormalities such as Osler-Weber-Rendu syndrome, which is considered to be caused by focal weakening of the blood vessel wall (4). Cheng (5) present a rare case of fatal hemoptysis due to bronchial aneurysm in a patient with active pulmonary tuberculosis. The patient in case 1 had a history of chronic pulmonary infection and bronchiectasis; however, many BAA patients, such as in case 2, do not have a pertinent history, which makes the diagnosis difficult. Pseudoaneurysm is a collection formed by a thrombus or fibrotic substance in its surrounding thrombus. Pseudoaneurysms may be caused by trauma, ruptured aneurysms, or postoperative anastomotic leakage (6).

For patients with BAA, the clinical manifestation depends on the location of the aneurysm and whether it is ruptured. BAA is located inside the mediastinum or inside the lungs. BAA in the mediastinum may show symptoms of compression, such as dysphagia (7, 8) or superior vena cava syndrome (9), or may enter adjacent structures causing ruptured aortic dissection (10). If the aneurysm is ruptured, the BAA inside the lung may cause hemoptysis or hematemesis. The most common symptom after aneurysm rupture is chest pain, followed by hemoptysis, back pain, epigastric pain, and shock. However, if the aneurysm remains intact, the BAA is usually incidentally found during a chest scan. In our hospital, there were two cases of BAA: one case which presented with hemoptysis secondary to bronchiectasis which was found on chest CT and the other case found after complaints of chest pain caused by a ruptured aneurysm. Bronchial arteriography revealed that the BAA in both cases was located inside the mediastinum.

Patients with true BAAs or pseudobronchial aneurysms can be treated with surgery or TAE. BAA resection, bronchial artery ligation, or total pneumonectomy can be performed *via* thoracotomy. Although the lesion can be completely resected, open surgery is more traumatic (11). Compared with surgical treatment, TAE is an effective minimally invasive treatment and is the first choice for BAA. The purpose of TAE is not only blocking the feeding vessels but also blocking the efferent branches to avoid retrograde filling of aneurysms. However, because the blood flow rate and coil transmission cannot be controlled, it is difficult to operate and may lead to embolism of other organs (11). Different methods such as direct embolization, combined film-coated stent isolation, and percutaneous puncture embolization should be selected according to the location and nature of the aneurysm.

If the proximal bronchial artery of the aneurysm is of sufficient length, endovascular coil embolization is preferred (7). However, the aneurysms in our two cases were mediastinal aneurysms, which were located at the opening and had a short neck, making it difficult to embolize the blood vessels. When the neck between the BAA and the aorta is short, the operation may be accompanied by distal material migration or insufficient embolization of the aneurysm. In case 1, to maintain a complete true aneurysm, coil and microsphere embolization was used, and the tumor cavity was blocked. To achieve satisfactory angiographic results, a thoracic aorta stent was not implanted. Moreover, placing the stent at this time may be an overtreatment. An additional stent in the thoracic aorta also increases the risk of spinal cord ischemia. Goh believes that placement of a stent-graft may make it impossible for the patient to receive endovascular treatment in the future. A more cautious approach may be to follow-up the patient and place the stent when needed in the future (12). Fu reported that five cases of aneurysms near the opening of the bronchial artery were treated with TAE. Hemoptysis disappeared in all patients. No special or severe complications occurred, and no patient experienced a recurrence during the follow-up (2). In case 2, the body of the pseudoaneurysm was larger, the neck was shorter, the risk of simple embolization was higher, and the patient was prepared for thoracic surgery for posterior mediastinal hematoma removal in the later stage; however, the operation had a risk of rebleeding. Therefore, implantation of a covered stent in the thoracic aorta through coil embolization was proposed combined with covered stent graft exclusion of the thoracic aorta. The stent covered the opening of the bronchial artery and isolated the aortic blood flow. Concurrently, PVA and coils were used to embolize the aneurysm to prevent the aneurysm from refilling. In this case, we successfully isolated a large bronchial aneurysm from the circulation using a thoracic aortic stent graft to block the inflow branch and used coil to embolize the outflow branch. During follow-up 9 months post-surgery, the mediastinal hematoma was almost completely absorbed. Long-term follow-up of the case and further experience of similar cases are needed to prove the feasibility of this technique. Sakai also reported similar cases using TAE combined with stent implantation to resolve the problem of short neck aneurysms (11, 13–18). Wang (19) reported an aortic stent combined with arterial embolization for the treatment of multiple BAAs with a diffuse bronchial artery and pulmonary artery fistula. Compared with simple embolization, the advantages of embolization and stent implantation include blocking the blood supply to the aneurysm, preventing distal material migration, and blocking the blood flow of potential collateral arteries from the thoracic aorta.

According to the literature, aneurysms with a short neck or no neck can be embolized with detachable coils (20). Mediastinal BAAs with short inflow vessels can be successfully treated with occluders in the patent ductus arteriosus (21). One report pointed out that percutaneous embolization is an option for pseudobronchial aneurysms that cannot be achieved by angiography (22, 23).

## Conclusion

Endovascular embolization has become the most commonly used and recommended technique, and different methods such as direct embolization, combined film-coated stent isolation, and percutaneous puncture embolization should be selected according to the location and nature of the aneurysm. The methods suitable for anatomical characteristics need to be selected according to the specific conditions of the lesion to achieve the ideal embolization effect, but it is also necessary to prevent over-treatment. Long-term follow-up and further experiences are necessary to evaluate the effectiveness of different treatments.

## Data Availability Statement

The original contributions presented in the study are included in the article/supplementary material, further inquiries can be directed to the corresponding authors.

## Ethics Statement

The studies involving human participants were reviewed and approved by the Ethics Committee of Fujian Provincial Hospital. The patients/participants provided their written informed consent to participate in this study.

## Author Contributions

J-LL, J-HZ, YT, D-DR, and R-LW performed the acquisition, analysis, and interpretation of the clinical data. J-LL, M-ZZ, and Y-YJ drafted the manuscript. S-JW, Y-FZ, S-LC, and X-RM provided critical revision of the manuscript. J-WL and Z-TF designed and supervised the study. All authors read and approved the final manuscript.

## Conflict of Interest

The authors declare that the research was conducted in the absence of any commercial or financial relationships that could be construed as a potential conflict of interest.

## Publisher’s Note

All claims expressed in this article are solely those of the authors and do not necessarily represent those of their affiliated organizations, or those of the publisher, the editors and the reviewers. Any product that may be evaluated in this article, or claim that may be made by its manufacturer, is not guaranteed or endorsed by the publisher.

## Abbreviations

BAA: bronchial artery aneurysm
TAE: transcatheter artery embolization
CT: computed tomography
CTA: computed tomography angiography
DSA: digital subtraction angiography
Hb: hemoglobin.
